# Differential Requirements of Two *recA* Mutants for Constitutive SOS Expression in *Escherichia coli* K-12

**DOI:** 10.1371/journal.pone.0004100

**Published:** 2008-12-31

**Authors:** Jarukit Edward Long, Nicholas Renzette, Richard C. Centore, Steven J. Sandler

**Affiliations:** 1 Department of Microbiology, Morrill Science Center IV N203, University of Massachusetts at Amherst, Amherst, Massachusetts, United States of America; 2 Molecular and Cellular Biology Graduate Program, Morrill Science Center, University of Massachusetts at Amherst, Amherst, Massachusetts, United States of America; University of Massachusetts Medical School, United States of America

## Abstract

**Background:**

Repairing DNA damage begins with its detection and is often followed by elicitation of a cellular response. In *E. coli*, RecA polymerizes on ssDNA produced after DNA damage and induces the SOS Response. The RecA-DNA filament is an allosteric effector of LexA auto-proteolysis. LexA is the repressor of the SOS Response. Not all RecA-DNA filaments, however, lead to an SOS Response. Certain *recA* mutants express the SOS Response (*recA^C^*) in the absence of external DNA damage in log phase cells.

**Methodology/Principal Findings:**

Genetic analysis of two *recA^C^* mutants was used to determine the mechanism of constitutive SOS (SOS^C^) expression in a population of log phase cells using fluorescence of single cells carrying an SOS reporter system (*sulAp*-*gfp*). SOS^C^ expression in *recA4142* mutants was dependent on its initial level of transcription, *recBCD*, *recFOR*, *recX*, *dinI*, *xthA* and the type of medium in which the cells were grown. SOS^C^ expression in *recA730* mutants was affected by none of the mutations or conditions tested above.

**Conclusions/Significance:**

It is concluded that not all *recA^C^* alleles cause SOS^C^ expression by the same mechanism. It is hypothesized that RecA4142 is loaded on to a double-strand end of DNA and that the RecA filament is stabilized by the presence of DinI and destabilized by RecX. RecFOR regulate the activity of RecX to destabilize the RecA filament. RecA730 causes SOS^C^ expression by binding to ssDNA in a mechanism yet to be determined.

## Introduction

Maintenance of genetic information is a priority for all organisms. The RAD51-RecA family of proteins plays a critical role in the repair of DNA through the production of a protein-DNA helical filament [1]. The function of both proteins are regulated (in part) by matching sets of evolutionary homologs {SRS2 and UvrD; BLM and RecQ [2]–[5]} or functional analogs {RAD52, RAD55, RAD57 and RecFOR [6]–[8]}. Eukaryotic cells have complex systems of proteins to detect DNA damage, transduce this information to block cell cycle checkpoints, increase the transcription of DNA repair genes and then repair the DNA {reviewed in [9], [10]}. RAD51 plays an important role in some of these processes through interactions with BRCA1 and BRCA2 {reviewed in [11], [12]}. In *E. coli*, RecA links these processes by its ability to detect and bind single-stranded DNA (ssDNA) produced by DNA damage to form a RecA-DNA helical filament. This structure then transduces the information that DNA damage exists in the cell by increasing the rate of LexA auto-proteolysis [13]. Decreasing the concentration of LexA, the repressor of the SOS Response, up-regulates a large set of genes (50 or more) that have both known functions (*i.e.*, DNA repair, mutagenesis and delay of cell division) and yet unknown functions [14]. Interestingly, as more SOS regulons are studied in diverse bacteria, the diversity of functions induced as part of SOS increases {*e.g.*, horizontal gene transfer of antibiotic resistance genes [15] and others reviewed in [16]} as do the diversity of antimicrobial compounds that induce SOS [17]–[19]. The RecA-DNA filament is also critical for DNA repair.

**Figure 1:**
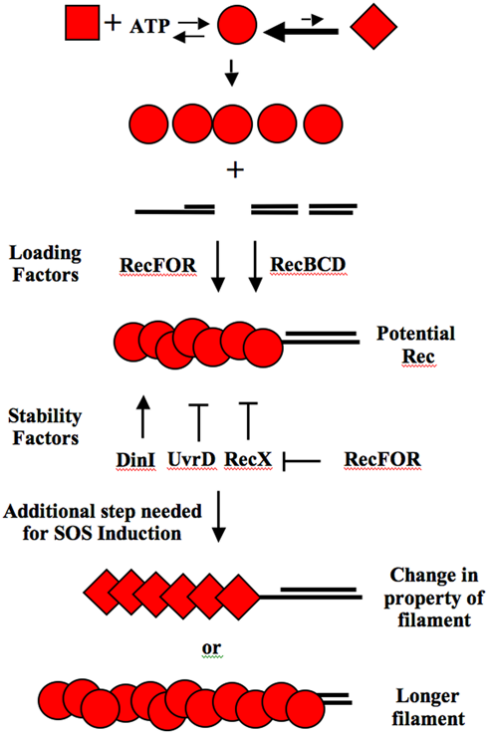
This figure shows models for how RecA interacts with proteins that load RecA onto ssDNA and or stabilize/destabilize the RecA-DNA filaments. Three forms of the RecA protein are shown. The square version is the RecA protein alone. It is not capable of binding to ssDNA. It must first bind ATP. RecA bound with ATP is pictured as the circular form. The circular version is capable of binding to ssDNA through the aid of RecFOR and RecBCD on their appropriate gapped or DSB substrates. The stability of the circular form of RecA on ssDNA is affected by DinI, RecX and UvrD as indicated. This circular form is competent for recombination, but not SOS Induction. Some other attribute is required for SOS induction. This could be the adoption of an activated form (portrayed as the diamond shape) and or a more extensive, longer filament of the circular form. Once the SOS inducing filament is formed, it is competent to interact with LexA and accelerate cleavage (see text for references).

RecA-DNA filaments exist in non-SOS inducing cells to recombinationally repair “broken” replication forks {reviewed in [20], [21]}. This is illustrated by the observation that in wild type cells 15% of populations of log phase cells have RecA-DNA filaments (as determined by RecA-GFP) while less than 0.3% of the cells are induced for the SOS Response [22]–[24]. Independently derived data also show that at least 15% of log phase cells are recombining their DNA in a RecA-dependent manner at any one time [25]. This suggests that there are many RecA-DNA filaments formed *in vivo* that do not lead to induction of the SOS Response. At least one difference between the requirements for recombination and SOS induction is that the ATPase activity of RecA, crucial for recombination, is not required for SOS induction [26]. This work further suggested that the ability to adopt an “extended conformation” may be important for SOS induction. This could mean that an SOS inducing RecA-DNA filament may adopt a special conformation or it may be longer or more stable than a filament poised for recombination (Figure 1). If the cell has the ability to distinguish when it is appropriate to allow the RecA-DNA filament to induce the SOS response, then it may be possible to find mutations in *recA* that constitutively express the SOS response (*recA^C^*) when it is not appropriate. Historically, many *recA^C^* alleles have been isolated {listed and reviewed in [27]}. A better understanding of why these mutants express SOS when they should not, may lead to a better understanding of how the cell regulates the function of RecA-DNA filaments.

One of the first *recA^C^* alleles to be isolated and characterized was *recA441*. This mutant was originally called *tif-1* for temperature-inducible filamentation [28]. It is now understood that this *recA* allele allowed temperature-dependent expression of the LexA-regulated division inhibitor, SulA [29]. Subsequent studies revealed that *recA441* has two missense mutations: E38K and I298V [30]. These two mutations were separated through recombination by transferring the *recA441* gene from *E. coli* K-12 into *E. coli* B/r [31]. The former (E38K) mutation is responsible for constitutive SOS expression and the latter (I298V) mutation is responsible for suppression of this phenotype at the permissive temperature. The single E38K mutation conferring the constitutive SOS expression phenotype was named *recA730*. This allele was also isolated independently using a plaque color assay and called *recA1211* [32]. Structural studies show that the *recA730* change (E38K) is located on the outside of the RecA-DNA helical filament [33]. Biochemically, RecA730 is able to better compete for ssDNA coated with Single-Stranded DNA Binding protein (SSB) than wild type RecA [34], [35]. Although this observation has been the basis for some models for the SOS^C^ phenotype (see below), other biochemically and genetically characterized mutants of *recA*, such as *recA803* (V37M) also have the ability to compete for SSB coated ssDNA better than wild type but do not display SOS^C^ expression (unpublished results). It has been recently shown that *recA730* can intra-genetically suppress the inability of *recA2201* K72R, an ATPase defective mutant, to induce the SOS Response after UV treatment [26]. It is thought that RecA730's ability to adopt an extended filament formation is critical for its ability to suppress this defect.

Other *recA^C^* alleles have been identified by mutagenizing a plasmid-encoded copy of *recA* and then over-expressing these mutant genes from a strong promoter. One *recA^C^* allele identified (and studied herein) has a phenylalanine codon at position 217 mutated to a tyrosine codon {now called *recA4142* (F217Y) [27]}. Structural studies show that this amino acid is located at the RecA monomer-monomer interface (in a different position from *recA730*) [33]. Biochemical analysis of RecA4142 shows that it has increased cooperativity when binding ssDNA relative to wild type [36].

One model to explain the ability of mutant RecA proteins to constitutively express the co-protease function is that these mutants bind to ssDNA to form a critical RecA-DNA filament in log phase cells in the absence of external DNA damage when wild type RecA does not. This assumes that there is adequate ssDNA is available in all cells. The site of the ssDNA has been hypothesized to be at the replication forks. As stated above, it has been shown that some *recA* alleles (*e.g.*, *recA730*) bind ssDNA in the presence of SSB better than wild type. If better ssDNA binding is all that is needed, then overproduction of the RecA^+^ protein should drive the equilibrium towards the bound state for all ssDNA in the cell and one should see high levels of SOS. This was tested and was not observed [37]. Therefore, *recA^C^* mutants must have additional capabilities that allow them to induce SOS when wild type does not.

There are several proteins that affect RecA's ability to load onto ssDNA and the stability of the filament (Figure 1). RecBCD and RecFOR provide two pathways for loading RecA onto ssDNA at Double-Stranded Breaks (DSBs) and gapped-DNA, respectively {reviewed in [38], [39]}. Sub-complexes of RecFOR (*i.e.*, RecFR and RecOR) can also affect the extent and stability of RecA-DNA filaments *in vitro* {[40]–[42] and reviewed in [43]}. Two SOS regulated genes that modulate RecA filament stability are *dinI* and *recX* {reviewed in [43], [44]}. DinI's role is complicated because it stabilizes RecA-DNA filaments at low ratios of DinI to RecA and destabilizes them at high ratios [45]–[47]. Specific interactions between RecA and DinI have been proposed [48]–[50]. RecA filaments grow in the 5′ to 3′ direction with subunits preferentially adding to the 3′ end and dissociating from the 5′ end [51]. Evidence supports the model that RecX destabilizes RecA-DNA filaments by either preventing growth of the filament at the 3′ end [52] or by binding to the middle of filaments, causing local instability and an increased number of 5′ ends from which RecA can dissociate [53]. An additional layer of regulation suggests that RecF(OR) antagonize RecX's ability to destablize RecA-DNA filaments *in vitro* [54]. *recA* and *recX* are co-expressed in a polycistronic mRNA and regulated by SOS [55].

Other proteins can also affect the number and or stability of RecA-DNA filaments. UvrD helicase can remove RecA from ssDNA *in vitro* and from certain types of arrested replication forks *in vivo* [5], [56]–[58]. XthA (Exonuclease III) does not affect RecA filaments directly, but rather affects the availability of substrates to which RecA can bind [59].

In this work, the *sulAp-gfp* reporter system [22] was used to monitor SOS^C^ expression in individual cells of two *recA^C^* mutants: *recA730* (E38K) and *recA4142* (F217Y). Mutational analysis of SOS^C^ expression in *recA730* and *recA4142* mutants suggests the two mutants have different requirements for SOS^C^ expression. SOS^C^ expression in the *recA4142* mutant is dependent on the initial level of transcription in the cell, proteins that help load RecA and proteins that stabilize (or destabilize) the filament. It is proposed that RecA4142 is loaded by RecBCD presumably at double-stranded ends that occur in log phase cells. DinI stabilizes this complex and RecX destabilizes it in the absence of RecFOR in minimal medium and in the presence of RecFOR in rich medium. Since no mutational dependence for *recA730* was established, no specific model for the DNA substrates that this protein binds, how it is loaded onto DNA or how it is stabilized or destabilized is currently offered.

## Results

**Figure 2:**
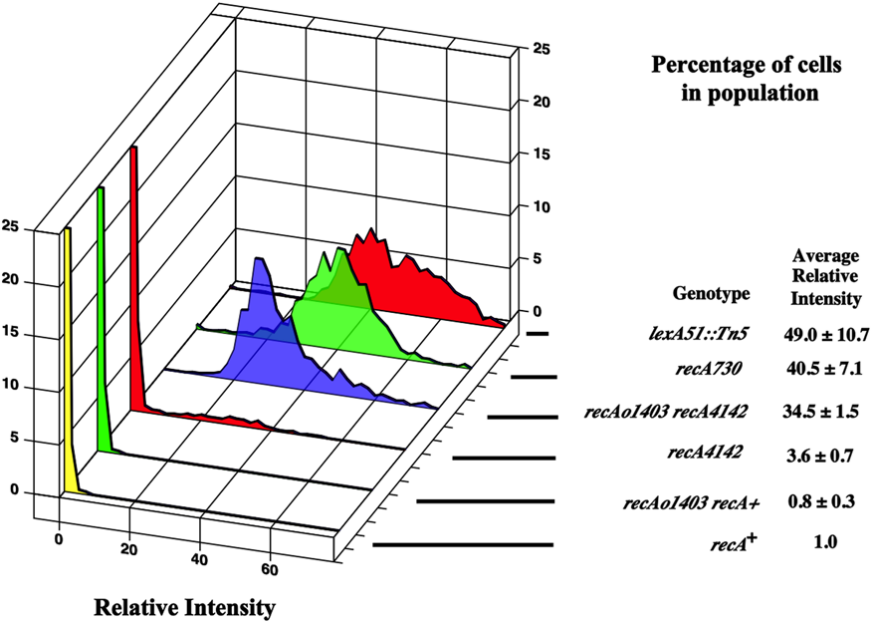
This figure shows the distributions of cells with different levels of constitutive SOS expression (detected as GFP fluorescence) expressed as the percentage of cells in the population. The graphs truncate the percentage of cells at 25%. The strains are in order from top of the graph to the bottom with the relevant part of the genotype in parentheses. Unless otherwise indicated, all strains were grown in minimal medium at 37°C with aeration. The strains are: SS1408 (*lexA51::Tn5*), SS4629 (*recA730*), SS4976 (*recAo1403 recA4142*), SS6013 (*recA4142*), SS6088 (*recAo1403 recA^+^*) and SS996 (*recA* ^+^).

To test if *recA^C^* mutants have high levels of SOS expression in all cells, the *recA^C^* alleles were combined with a *sulAp-gfp* transcriptional fusion inserted at the *att*λ site and were measured for relative fluorescence intensity as previously described [22]. The *sulA* promoter is induced early during SOS expression [14] and is a robust measure of SOS expression showing increases of 60–125 fold depending on the reporter system {reviewed in [22], [44]}. It is also a sensitive measure of SOS expression, being induced by very low doses (5 joules) of UV irradiation that have negligible effect on the survival of the population [24], [60]. Additionally, all strains used in this study have the *sulB103* allele (this is an allele of *ftsZ*) that suppresses SOS cell division inhibition [29]. For analysis, cells are grown in minimal medium into mid-exponential phase and placed on an agarose pad on a microscope slide where images of three fields of 200–300 cells each are taken from three different experiments (nine fields altogether). These cells are then measured for their total Relative Fluorescence Intensity (RFI) against fluorescent beads and then are normalized against a wild type cell containing *sulAp-gfp*. The RFI of the population of cells from all three experiments (typically 1000–3000 cells) are combined and binned according to their RFI. The percentage of cells with a particular RFI is calculated and plotted. The average RFI for each experiment is also calculated. The average for the three experiments and their uncertainties is reported next to the plots in the Figures. Figure 2 shows the distribution of a *lexA51::Tn5* (null allele) strain. These cells form a normal distribution with an average RFI of 49±10. Figure 2 also shows *recA* ^+^ cells that have an average RFI of 1. Very few wild type cells (less than 1%) have a total RFI more than six-fold above the average wild type level. The six-fold level is a convenient cut-off for cells that are not constitutive for SOS expression {see [22] for previous considerations for this argument}.

### Initial characterization of *recA^C^* single mutants

Figure 2 shows that *recA730* (E38K) cells have a normal distribution and an average RFI of 40±7 units. More than 99% of the cells have a total RFI six-fold above wild type. The average RFI is not significantly different from a *lexA51::Tn5* null mutant. Cells containing *recA4142* (F217Y) had an average RFI of about 3.6±0.7. This is nearly 10-fold less that of a *recA730* strain. Approximately 8% of these cells had a total RFI that was six-fold greater than the average wild type cell. Figure 2 shows that the distribution of *recA4142* cells was continuous with a long tail of cells with higher levels of expression. Therefore, these constitutive *recA* alleles have different properties in terms of the levels of SOS^C^ expression and the percentage of cells expressing SOS.

### *recAo1403* increase SOS^C^ expression in *recA4142* mutants

Only 8% of the *recA4142* cells had high SOS^C^ expression. Since *recA4142* was characterized to have high levels of SOS^C^ expression when expressed from a plasmid (see above), it is possible that the concentration of RecA4142 did not allow a majority of the cells to reach a critical threshold needed to display high levels of SOS^C^ expression. This suggests that increasing the level of transcription of the *recA4142* gene 2–3 fold with a *recAo1403* mutation in the operator/promoter region of *recA* may increase the proportion of cells with high level of SOS^C^ expression. Figure 2 shows that all *recAo1403 recA4142* cells have high SOS^C^ expression with an average RFI of 34.5±1.5. *recAo1403 recA* ^+^ cells have a profile and average RFI like wild type cells (Figure 2). It is also possible that the *recA4142* mutation destabilizes the RecA protein and this is the reason why increased levels of transcription are needed to achieve SOS^C^ expression. However, western blots of *lexA3* strains with *recA* ^+^ and *recA4142* show that these strains have equal amounts of RecA protein (data not shown).

From these results, it is concluded that the level of RecA4142 in some cells is not quite high enough to bind ssDNA available in cells to provide SOS^C^ expression. Its ability in some cells to induce SOS expression may be due to stochastic fluctuations in levels of RecA4142 expression or amounts of ssDNA. However, if the level of transcription is increased 2–3 fold, this condition is then sufficient to allow RecA4142 to bind ssDNA in every cell and thus 100% show SOS^C^ expression. These results are consistent with the idea that the limiting step in SOS^C^ expression is the formation of a RecA-ssDNA helical filament capable of co-protease activity. This is dependent on the initial concentration of RecA and its ability to bind its substrate.

### The dependence of RecA loading factors on SOS^C^ expression

*In vivo* RecA requires either the RecBCD enzyme to load onto ssDNA generated at a DSB or the RecFOR proteins to load onto gapped DNA (see above). In both cases, these proteins allow RecA to overcome inhibition by SSB that may coat the ssDNA. Whether the *recA^C^* alleles require RecBCD or RecFOR for loading may yield an additional clue as to their actual substrate. To test if the absence of the *recBCD* and *recFOR* genes have effects on SOS^C^ expression, *del*(*recBCD*)::*cat* and *recF4115* were introduced into *recA730* and *recAo1403 recA4142* strains. The notation *recF4115(OR)* will be used in the next several sections to indicate that these experiments have also been done with *recR* and *recO* mutations. These data, however, will not be shown due to their redundant nature with the *recF4115* data.

**Figure 3:**
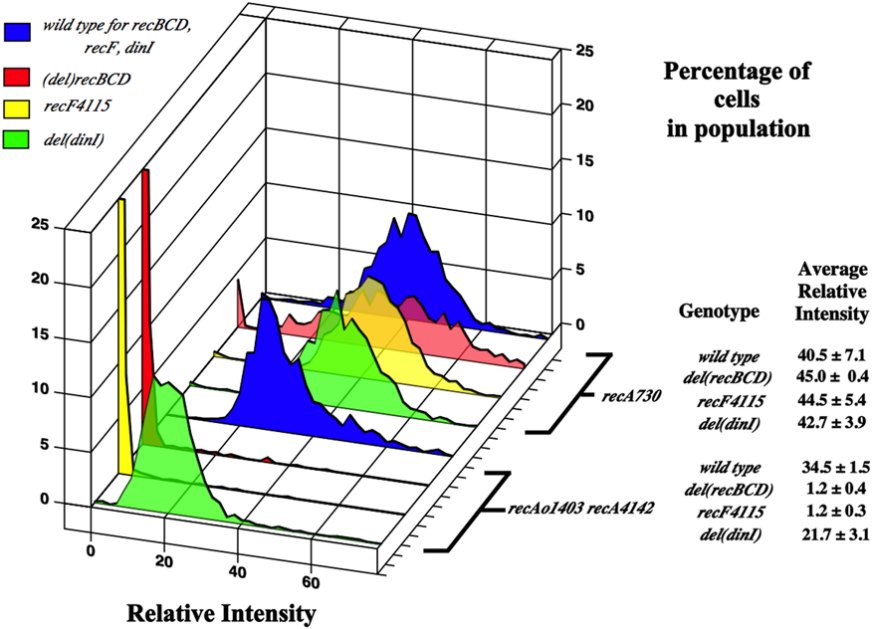
Same as for Figure 2. SS4629 (*recA730*), SS6044 (*recA730 del(recBCD)::cat*), SS4645 (*recA730 recF4115*), SS5316 (*recA730 del(dinI)*), SS4976 (*recAo1403 recA4142*), SS6023 (*recAo1403 recA4142 del(recBCD)::cat*), SS4696 (*recAo1403 recA4142 recF4115*), SS5315 (*recAo1403 recA4142 del(dinI)*).

Figure 3 shows that when *del(recBCD)::cat* or *recF4115(OR)* mutations are added to a *recA730* strain, they have little effect on the total relative intensity of the strain or the percentage of cells expressing SOS. Interestingly, however, the *del(recBCD)::cat* mutation causes a broadening of the distribution. This is not seen with the *recF4115(OR)* mutant.

The *del(recBCD)::cat* and *recF4115(OR)* mutations had a much different effect in the *recAo1403 recA4142* strain. In each case the average RFI of the strain decreased to nearly wild type levels (Figure 3). This decrease could be complemented in its respective strain by the addition of a plasmid with either the *recF(OR)* genes or the *recBCD* genes (data not shown).

It is concluded that unlike *recA730*, SOS^C^ expression in the *recA4142* mutant is dependent on both the RecBCD and RecFOR proteins. It was unexpected that both the *del(recBCD)::cat* and *recF4115(OR)* mutations would have the same effects since they are involved in different pathways of loading. At best, one would have predicted an additive effect if both DSBs and gaps were involved. Experiments shown below suggest that RecFOR's role in this process is to antagonize RecX.

### *dinI* is required for constitutive SOS expression in *recA4142* mutants

DinI has been shown, *in vivo* and *in vitro*, to stabilize RecA filaments when in low ratios of DinI to RecA (see above). To test if DinI stabilizes the RecA-DNA filament for SOS^C^ expression, a *dinI* deletion was combined with *recA730* or *recAo1403 recA4142*. Figure 3 shows *del(dinI)* has no effect on the levels of SOS^C^ expression in the *recA730* mutant. Unlike the *recA730* mutant, a 30% decrease in SOS^C^ expression (the average RFI) was seen when *del(dinI)* was combined with *recAo1403 recA4142* (Figure 3). *del(dinI)* causes a shift of the entire distribution towards the lower end of the scale. This suggests that the RecA-DNA filaments in the mutant are destabilized across the entire population in an even manner.

### In minimal medium, *del(recX)* has little effect on the SOS^C^ expression in *recA730* and *recA4142* strains

**Figure 4:**
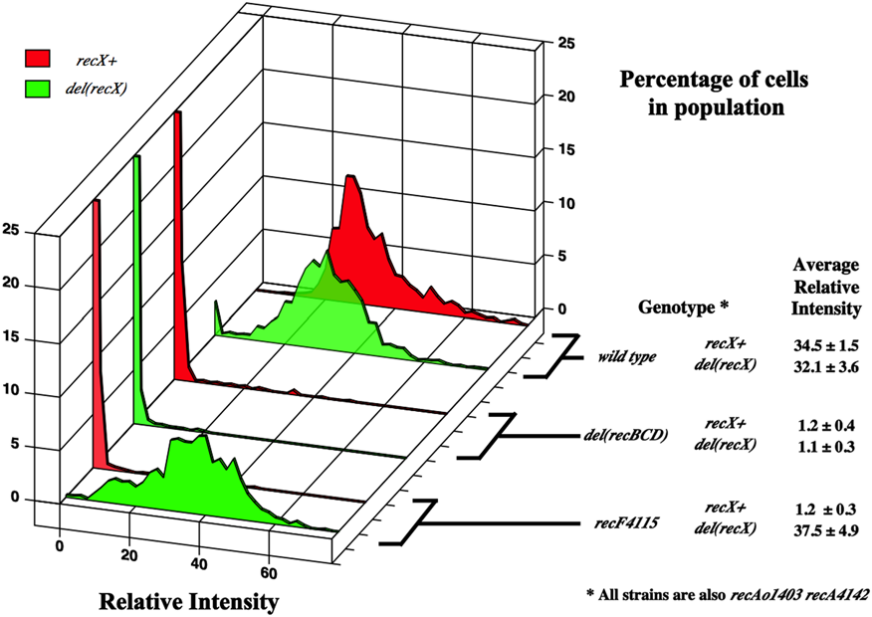
Same as for Figure 2. SS4976 (*recAo1403 recA4142*), SS5312 (*recAo1403 recA4142 del(recX)*) SS6023 (*recAo1403 recA4142 del(recBCD)::cat*), SS6048 (*recAo1403 recA4142 del(recBCD)::cat del(recX)*), SS4696 (*recAo1403 recA4142 recF4115*), SS5394 (*recAo1403 recA4142 recF4115 del(recX)*).

As mentioned above, RecX has been shown to destabilize RecA filaments *in vitro*. Other observations suggest that RecX interacts with the C-terminal residues of RecA [47], [61]. It was predicted that *recA730* and *recA4142* mutants would show no *recX*-dependence in minimal medium because it has been shown, using *recA-gfp*, that the ability to detect a *recX*-dependent change in the number of RecA-GFP foci is only seen in rich medium [47]. To test if the absence of *recX* would increase the level of SOS^C^ expression in strains containing *recA730* or *recA4142*, *del(recX)::cat* was introduced. The average RFI of *recA730* and *recA4142* strains grown in log phase in minimal medium did not change significantly with the addition of a *recX* mutation (Figure 4 and data not shown). It is possible that a *recX*-dependence could be seen if the strains were grown in rich medium. This will be tested below after the *recA^C^* mutants are initially characterized in rich medium.

### RecFOR antagonize the destabilizing effects of RecX on RecA4142 filaments

As mentioned above, it was surprising that a decrease in SOS^C^ expression was seen when mutations removed either the *recBCD* or the *recF(OR)* genes in *recAo1403 recA4142* cells. At least two models could suggest how this might happen. The first model suggests that RecBCD and RecFOR form a hybrid pathway for loading RecA4142 on DNA [62]. Alternately it is possible that RecBCD is important to load RecA on DNA and that RecFOR is necessary to protect the RecA4142 filament from RecX's ability to destabilize the RecA filament. This latter idea is based on experiments that show that RecFOR are needed to load RecA onto ssDNA coated with SSB in the presence of RecX and that RecF (but not RecOR) can physically interact with RecX [54]. This latter model predicts that the addition of a *recX* mutation to *recAo1403 recA4142 recF4115(OR)* cells should rescue the low level of SOS^C^ expression. Importantly, however, this should not occur in the *recAo1403 recA4142 del(recBCD)::cat* derivative. The appropriate mutants were constructed. Figure 4 shows that the *del(recX)* mutation rescues the SOS^C^ expression in the *recAo1403 recA4142 recF4115* strain. This was also true for the *recO* and the *recR* derivatives. Figure 4 also shows that addition of *del(recX)* to the *del(recBCD)::cat* derivative does not restore SOS^C^ expression.

It is also possible that RecX's inhibition of SOS^C^ expression in the *recAo1403 recA4142* strain is due to the fact that *recX*, in addition to *recA4142*, is being transcribed at higher levels. To test this, the identical *recF(OR)*, *recX* and *recA4142* mutant strains were constructed, but this time with *recAo* ^+^ instead of *recAo1403*. The *recAo* ^+^ *recA4142 recX^+^ recF^+^* strain has an average level of SOS^C^ of 3.6±0.7. Introduction of a *recF(OR)* mutation reduced this value to nearly background levels (1.3±0.5) and then this is restored by a *recX* mutation (5.7±2.3). Therefore, a similar pattern of SOS^C^ expression is seen between the *recAo* ^+^ and the *recAo1403* set of strains. Therefore the ability of RecX to decrease the level of SOS^C^ expression in the *recAo1403 recA4142* mutant is not due to increased level of expression in the *recAo1403* strain compared to the *recAo^+^* strain.

It is concluded that in *recAo1403 recA4142* cells, the RecBCD enzyme is crucial to load the mutant RecA protein at presumably DSBs and that RecFOR are vital to stabilize the RecA-DNA filaments by antagonizing the destabilizing effects of RecX. It is noteworthy that this effect of *recX* occurred when the cells were grown in minimal medium.

### *recA4142* cells grown in rich medium have lower levels of SOS^C^ expression than cells grown in minimal medium

The data above showing that *recFOR* was required for SOS^C^ expression if RecX was present suggests that RecX destabilized the *recA4142* filaments. Testing the single *recX* mutant in minimal medium above, however, showed no effect. As indicated, this was expected since the ability to detect a *recX*-dependent change in the number of RecA filaments was dependent on rich medium [47]. To begin to test this, the *recA4142* strain was characterized for SOS^C^ expression in rich medium.

**Figure 5:**
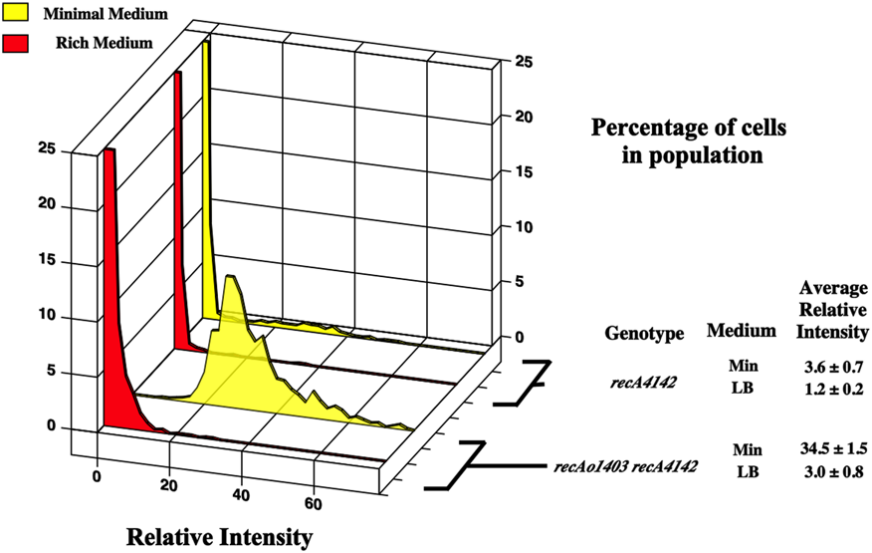
Same as for Figure 2. SS6013 (*recA4142*) minimal, SS6013 (*recA4142*) rich, SS4976 (*recAo1403 recA4142*) minimal, SS4976 (*recAo1403 recA4142*) rich.

Figure 5 shows that the *recA4142* mutant had 3-fold decreased SOS^C^ expression when grown in rich media compared to minimal medium. This was unexpected. To test if the amount and or binding capacity of the RecA4142 was still limiting in rich medium as it was in minimal medium, *recAo1403 recA4142* mutants were measured. Figure 5 showed that *recAo1403* only increased the level of SOS^C^ expression 2–3 fold. It did not produce the large 10-fold increase seen in minimal medium. This increase in SOS^C^ expression was compatible with the expected increase in transcription for the *recA* operator mutation suggesting that the amount of RecA4142 may still be limiting for SOS^C^ expression in rich medium or the DNA substrate. The level of expression and or distribution of SOS^C^ expression of *recA730* cells was not dependent on the type of medium.

### RecX destabilizes the RecA4142 filaments

**Figure 6:**
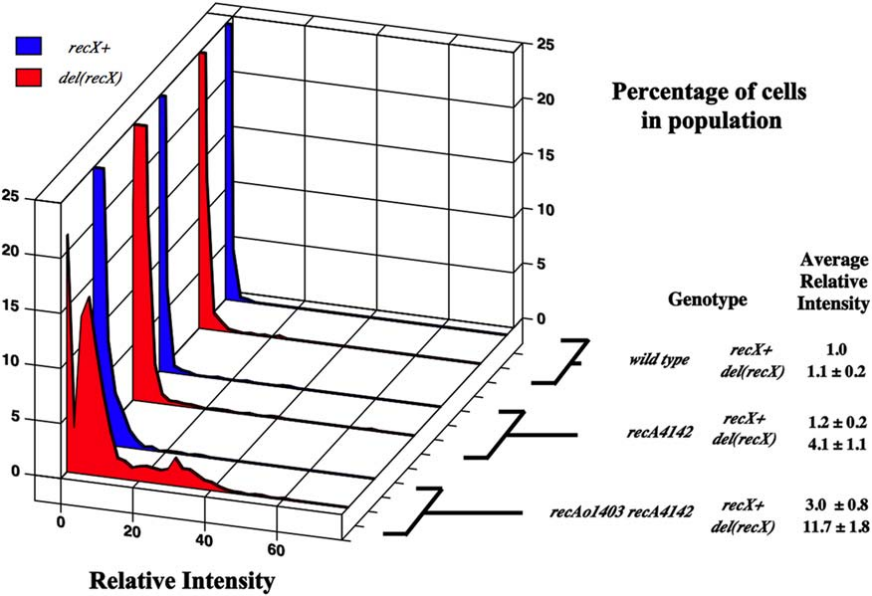
Same as for Figure 2. All grown in rich medium: SS996 (*recA* ^+^), SS6080 (*del(recX)*), SS6013 (*recA4142*), SS6019 (*recA4142 del(recX)*), SS4976 (*recAo1403 recA4142*), SS5312 (*recAo1403 recA4142 del(recX)*).

Since the *recA^C^* mutants were characterized in rich medium, it is now possible to ask whether RecX destabilizes the RecA filaments in these strains. If so, one would expect that mutating *recX* should increase the amount of SOS^C^ expression across the population. Figure 6 shows that *del(recX)* in *recA4142* and *recAo1403 recA4142* strains increases the average RFI about 3–4 fold in each case. It was also tested if *del(recX)* would increase the level of SOS^C^ expression of *recA730* cells by the creation of a *recA730 del(recX)* double mutant. This double mutant did not show increased levels of SOS^C^ expression (data not shown).

It is concluded that RecX can destabilize *recA4142* filaments. This destabilization is *recFOR*-independent (thus different from that described above in minimal medium). It is not clear if RecX has the ability to affect *recA730* filaments since these cells already seem to be at the highest level of SOS^C^ expression.

### Exo III opposes constitutive SOS expression in *recA4142*

*xthA* is the structural gene for Exonuclease III. It has been shown that *xthA* mutants have about three times as many RecA-GFP foci as wild type cells when grown in minimal medium in exponential phase [59]. The majority of these foci are thought to occur at DSBs where RecBCD helps to load RecA since *recB* mutations decrease the number of foci dramatically and *xthA* mutants have more double strand ends as measured by pulse-field gel electrophoresis [59]. Since SOS^C^ expression by *recA4142* is *recBCD*-dependent, it is possible that RecA4142 loads at double-strand ends normally processed by Exo III. If true, then the increase in the number of RecA-GFP foci of an *xthA* mutant should be medium dependent (like the SOS^C^ expression in *recA4142* mutants). There should also be an increase in the number of SOS^C^ cells in a population of *recA4142 del(xthA)* mutants grown in minimal medium and the increase should be *recBCD*-dependent.

**Figure 7:**
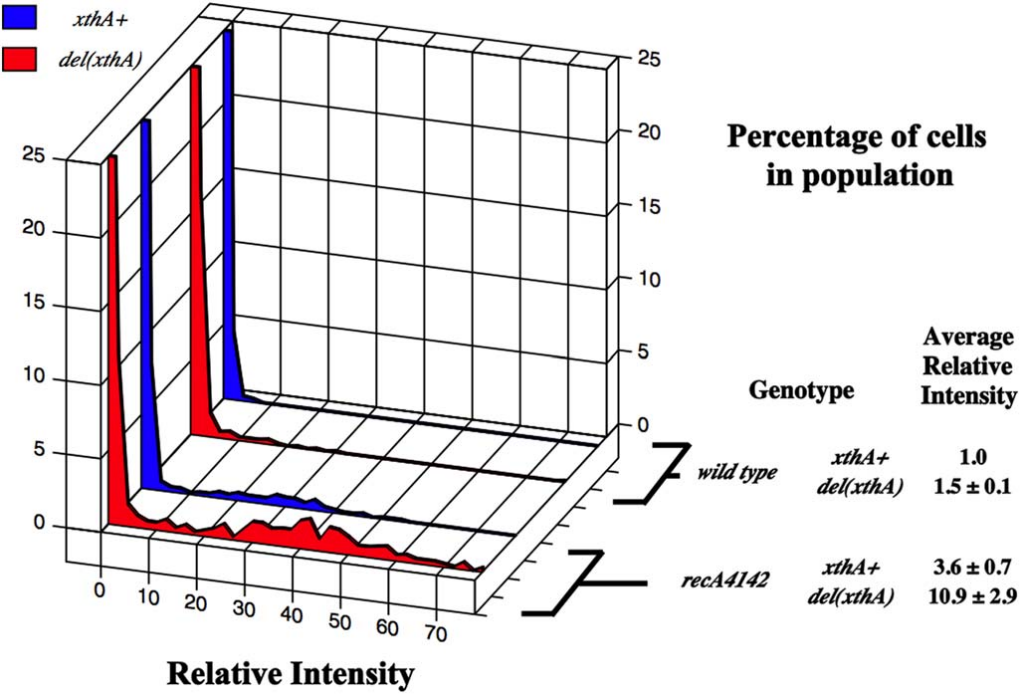
Same as for Figure 2. SS996 (*recA* ^+^ *xthA* ^+^), SS4857 (*recA* ^+^ *del(xthA)*), SS6013 (*recA4142*), SS6094 (*recA4142 del(xthA)*).

The first prediction was tested by growing SS3085 {*recA-gfp xthA* ^+^} and SS4560 {*recA-gfp del(xthA)*} in log phase in Luria broth and comparing the number of foci. These two strains showed distributions of RecA-GFP foci that were nearly identical (data not shown). The *recA4142* mutant was then combined with an *del(xthA)* mutation to test their level of SOS^C^ expression. Figure 7 shows that removal of *xthA* caused a three fold increase in the average RFI of the *recA4142* strain (minimal media). The *xthA recA^C^* strains were then combined with a *recBCD* mutation and the level of SOS^C^ expression decreased back to the level of the *recA^C^* mutant alone (data not shown). These data are consistent with the idea that in *del(xthA)* mutants, RecA4142 produce SOS^C^ expression when loaded at a double-stand end in a RecBCD-dependent manner.

## Discussion

RecA and LexA regulate SOS expression in response to DNA damage. It has been known that the formation of a RecA-DNA filament is crucial to sensing DNA damage inflicted by externally added DNA damaging agents (*i.e.*, UV irradiation or mitomycin C) and initiating the SOS Response. It has only become recently appreciated that RecA-DNA filaments form in log phase cells in response to spontaneous DNA damage caused by standard cellular metabolism and that these do not induce the SOS Response. Thus, the cell has some way to discriminate between these two situations. *recA^C^* alleles may be defective in this regulation as they promote the SOS functions in the absence of external DNA damage. Detailed analysis of two *recA^C^* alleles at the single cell level for SOS expression revealed that they have differential requirements for loading and stability factors. This further suggests, but does not prove, that they may be binding different DNA substrates. It is possible that RecA730 is able to bind the same substrate as RecA4142, but due to its mutation, it can do so in a manner different than RecA4142.

The working hypothesis at the beginning of this study suggested that the different *recA^C^* alleles should have the same requirements for SOS^C^ expression because their biochemical characterization, better binding to ssDNA and better cooperativity of binding to ssDNA, seem to indicate a similar mechanism for the SOS^C^ phenotype. It was therefore surprising that the two mutants had very different requirements for SOS^C^ expression. This suggests that other *recA^C^* mutants might also vary in their requirements for SOS^C^ expression. For instance another *recA^C^* allele, *recA4161* (a mutant where the last 17 amino acids of *recA* have been deleted), is like *recA4142* in that its SOS^C^ expression is limited initially by its level of expression and requires DinI for maximum levels of SOS^C^ expression, but it is like *recA730* in that its SOS^C^ expression is not dependent on RecBCD, RecFOR or RecX (unpublished results).

It is striking that a 2–3 fold change in the level of transcription of *recA4142* could push the number of cells in a population expressing SOS from 8% to 100%. A simple chemical equilibrium model can be invoked to explain this data. Remembering that to induce SOS, RecA needs to bind to ssDNA to make the RecA-ssDNA filament. Increasing the amount of either substrate (the RecA or the ssDNA) shifts the equilibrium towards complex or filament formation (and SOS expression). This assumes that loading and stability factors are not rate limiting. The previous report on *recA4142* showing that when it was expressed from a plasmid it had high level of SOS^C^ expression suggests that other *recA^C^* mutants identified on plasmids may also be limited for SOS^C^ expression when placed in single copy on the chromosome.

A key piece of information for understanding the mechanism of SOS^C^ expression is the type of DNA substrate bound by the RecA^C^ protein. It is clear that the loading of RecA4142 is RecBCD-dependent. This suggests that RecA4142 binds to a double-strand end with the help of RecBCD. The requirement of RecFOR for SOS^C^ expression in *recAo1403 recA4142* strains in the presence of RecX is consistent with the observation that all three proteins are required to allow assembly of RecA on ssDNA coated with SSB in the presence of RecX [54]. It is notable that the ability of RecX to inhibit SOS^C^ expression in the absence of RecFOR is dramatic (equal to the absence of RecBCD) and occurs in minimal medium. This is in contrast to the more subtle destabilizing effect RecX has in rich medium that is independent of RecFOR.

This study was initiated to try to understand why most RecA filaments that exist in log phase cells do not induce the SOS response. These studies suggest that wild type cells may have a two-tiered mechanism that prevents spurious SOS induction when RecA filaments are assembled for normal housekeeping events (*i.e.*, stabilization and fixing of some types of replication fork damage). The first tier operates at the level of maintaining the concentration of RecA such that it is just high enough to bind appropriate substrates (*i.e.*, ssDNA at stopped replication forks), but not high enough to bind inappropriate substrates that may exist in the cell. This may be why *recAo1403* is required to show full SOS^C^ expression in a population of *recA4142* cells. It is possible that RecA storage structures give the cell yet another method to tightly regulate the effective, available concentration of RecA [24]. The lack of additional SOS^C^ expression when RecA is overproduced {*i.e.*, *recAo1403* and [37]} or in *xthA* mutants [59] when RecA may be binding to inappropriate sites, suggests a second level of prevention. This second layer of regulation could take several forms. One form could be the removal of RecA from DNA by proteins like UvrD (see above for references). Another could be the action of proteins like RecX that selectively destabilize RecA-DNA filaments. It should be noted that both *uvrD* and *recX* are SOS regulated genes and so once SOS induction has occurred, their increased expression would serve to reduce RecA filament formation and reset the system. Additional proteins may also be involved. It is possible that some of the non-DNA repair SOS constitutive mutants identified by O'Reilly and Kreuzer [63] may be candidates for these proteins. In this scenario, SOS induction finally occurs when the amount of RecA-DNA exceeds a certain threshold level that saturates the mechanism(s) in this second layer. In this way, the cell can measure the amount of DNA damage regardless of its origin (spontaneous or external). It is plausible that this second layer of regulation acts by preventing RecA from adopting the special or longer conformation necessary for SOS induction and that *recA^C^* mutations like *recA730* and *recA4142* are immune to, or overcome, this regulation. Lastly, while this two-tiered model explains well the data obtained with *recA4142*, it does not explain why *recA730* does not need a *recAo1403* mutation to boost its initial concentration. One possible explanation for this is that RecA730 already binds ssDNA much better than wild type or RecA4142 and can adequately shift the equilibrium in the direction of complex formation (see above for references).

There are two paradoxical observations presented in this work. The first is that almost all the SOS^C^ expression in *recAo1403 recA4142* cells is dependent on the RecBCD enzyme. Since it is thought that the RecBCD enzymes loads RecA only at a double strand end produced at a DSB, this suggests that there is a DSB in every cell. If this were true, then *recAo1403 recA4142 recBCD* mutants should not be viable since repair of DSBs is essential for growth [64]. This paradox is also seen where *xthA* mutants have three-fold more RecA-GFP loading events than *xthA* ^+^ cells and two-fold more double strand ends than wild type; and yet *xthA recBCD* mutants are also viable [59]. While there is no *in vitro* data to support this proposal, it is possible that RecBCD loads RecA at some DNA substrate that exists in cells that are not double strand ends of DNA. A second idea is to explain this paradox is that RuvAB can reverse an arrested replication fork to produce a double-strand end [65]. It is possible that RecBCD loads RecA4142 onto this substrate. If this were true, then replication fork reversal would have to occur very often in *recAo1403 recA4142* mutants to explain the observation that all cells are SOS^C^. A third alternative to the above two models is that RecA4142 creates DNA damage by not properly processing recombinational intermediates. This idea has been used to explain the SOS^C^ expression of the recombination deficient *recA N99* mutants [66]. This idea is not supported, however, by the fact that *recA4142* mutants are as recombination proficient and UV resistant as wild type (Table 1). The second paradox is that there appears to more RecBCD-dependent RecA4142 loading events in minimal medium than in rich medium grown cells. This observation is counter-intuitive because it is thought that there is more on-going DNA replication in rich medium grown cells (where multiple rounds of chromosomal replication are occurring concurrently) than in minimal medium and this would lead to more instances where DNA replication forks might collapse, creating more double strand ends where RecBCD could load RecA. Additionally, this does not agree with previous findings that there are more RecB-dependent RecA-GFP foci in rich medium than in minimal medium [24]. It is not clear if these paradoxes are due to separate or related mechanisms. Further work will be necessary to unravel these complexities.

**Table 1:** Summary of phenotypic analysis of *recA* mutants used in this study.

Strain	*recAo*	*recA*	% Recombinants per 100 donors	% Surviving 5 J/m^2^ of UV	SOS expression ratio after 5 J/m^2^ of UV
SS996	+	+	1.09±0.26	80.0±3.8	8.7±2.8
SS391	+	*938::cat*	0.0006±0.0002	<0.001	ND a
SS4629	+	*730*	1.50±0.14	78.0±2.0	ND
SS6013	+	*4142*	1.79±0.34	87.8±6.3	11.1±1.8
SS4976	*1403*	*4142*	1.11±0.28	83.1±5.6	ND

a ND is Not Determined because the cells are already fully induced for SOS expression.

## Materials and Methods

### Bacterial strains

All bacterial strains used in this work are derivatives of *E. coli* K-12 and are described in Table 2. The protocol for P1 transduction has been described elsewhere [67]. All P1 transductions were selected on 2% agar plates containing either minimal or rich media. Where appropriate plates also contained the following antibiotics at these final concentrations: tetracycline 10 µg ml^−1^, chloramphenicol 25 µg ml^−1^ or kanamycin 50 µg ml^−1^. All transductants were purified on the same type of media on which they were selected. When necessary the *recA^C^* alleles (single and double mutants) were placed on the chromosome in the place of *recA* ^+^ as previously described (see below). Table 1 shows the characterization of these mutants for their survival to UV irradiation, ability to inherit markers during conjugation and the ability to induce the SOS response. Specific protocols for these tests have been previously described [68], [69]. Oligonucleotide primers used in this work are shown in Table 3.

**Table 2:** Strains used in this work.

Strain	*ygaD*	*recAo*	*recA*	*recX*	*recBCD*	*recF*	*attλ*	Other relevant genotype	Origin of reference
AB4117	*+*	*+*	*+*	*+*	*+*	*+*	*+*	*alaS5*	*E.coli* Stock Center
CAG18491	*+*	*+*	*+*	*+*	*+*	*+*	*+*	*argE+*	*E.coli* Stock Center
CAG18642	*+*	+	+	*+*	*+*	*+*	+	*zfj-3131::Tn10*	*E.coli* Stock Center
CF3032	*+*	*+*	*+*	*+*	*+*	*+*	*+*	*argA::Tn10*	Mike Cashel
DE391	*+*	*+*	*730*	*+*	*+*	*+*	*+*	*srlC300::Tn10*	H. Echols
JC13509	*+*	*+*	*+*	*+*	*+*	*+*	*+*		Lab Stock
JC17335	*+*	+	*730*	*+*	*+*	*+*	+		Lab Stock
JC18825	*+*	*+*	*+*	*+*	*+*	*4115*	*+*	*tnaA300::Tn10*	[71]
KM78	*+*	*+*	*+*	*+*	*cat* j	*+*	*+*		K. Murphy
SS391	*+*	+	*938::cat*	*+*	*+*	*+*	*+*		Lab Stock
SS775	*+*	*+*	*+*	*+*	*+*	*+*	*+*	*lexA3 malE::Tn10-9*	Lab Stock
SS996	*+*	+	+	*+*	*+*	*+*	Ω*gfp* j		[22]
SS1408	*+*	*+*	*+*	*+*	*+*	*+*	Ω*gfp*	*lexA51::Tn5*	[22]
SS1426	*+*	*+*	*+*	*+*	*+*	*4115*	Ω*gfp*	*tna300::Tn10*	[22]
SS2228	*+*	+	*+*	*+*	*+*	*+*	+	*zfj-3131::Tn10 alaS5*	CAG18642→AB4117 c
SS3085	*kan*	*1403*	*4155,4136*	*+*	*+*	*+*	+		[59]
SS4195	*+*	*+*	*730*	*cat* j	*+*	*+*	Ω*gfp*		SS4971→SS996 h
SS4421	*+*	*+*	*+*	*+*	*+*	*+*	+	*del(dinI)100::kan*	[72]
SS4560	*kan*	*1403*	*4155,4136*	*+*	*+*	*+*	+	*del(xthA)200::frt*	[59]
SS4626	*+*	*+*	*+*	*+*	*+*	*+*	Ω*gfp*	*zfj-3131::Tn10 alaS5*	SS2228→SS996 c
SS4629	*+*	*+*	*730*	*+*	*+*	*+*	Ω*gfp*		JC17335→SS4626 ^?^
SS4645	*+*	*+*	*730*	*+*	*+*	*4115*	Ω*gfp*	*tnaA::miniTn5 cat*	SS1876→SS4629 d
SS4696	*kan*	*1403*	*4142*	*+*	*+*	*4115*	Ω*gfp*	*tnaA300*::*Tn9*	SS1876→SS4976 d
SS4857	*+*	*+*	*+*	*+*	*+*	*+*	Ω*gfp*	*del*(*xthA*)*200::frt*	SS4555 g
SS4976	*kan*	*1403*	*4142*	*+*	*+*	*+*	Ω*gfp*		SS4973→SS996 i
SS5003	*+*	+	*+*	*+*	*+*	*+*	Ω*gfp*	*del(dinI)100::kan*	SS4421→SS996 b
SS5312	*kan*	*1403*	*4142*	*cat*	*+*	*+*	Ω*gfp*		SS5303→SS996 d
SS5313	*+*	+	*+*	*+*	*+*	*+*	Ω*gfp*	*del(dinI)200::frt*	SS5306 g
SS5315	*kan*	*1403*	*4142*	*+*	*+*	*+*	Ω*gfp*	*del(dinI)200::frt*	SS4973→SS5313 b
SS5316	*+*	+	*730*	*+*	*+*	*+*	Ω*gfp*	*srlC300::Tn10 del(dinI)200::frt*	DE391→SS5313 c
SS5394	*kan*	*1403*	*4142*	*cat*	*+*	*4115*	Ω*gfp*	*tnaA300::Tn10*	JC18825→SS5312 c
SS5438	*+*	+	*+*	*+*	*+*	*+*	+	*argE+*	CAG18491→JC13509 c
SS5446	*+*	+	*+*	*+*	*+*	*+*	+	*argA::Tn10*	CF3032→SS5438 c
SS6013	*kan*	+	*4142*	*+*	*+*	*+*	Ω*gfp*		SS6009→SS996 i
SS6019	*kan*	+	*4142*	*cat*	*+*	*+*	Ω*gfp*		SS6018→SS996 i
SS6020	*+*	*281*	*+*	*+*	*+*	*+*	Ω*gfp*	*srlC300::Tn10*	MV1138→SS996 c
SS6023	*kan*	*1403*	*4142*	*+*	*cat*	*+*	Ω*gfp*		KM78→SS4976 d
SS6044	*+*	+	*730*	*+*	*cat*	*+*	Ω*gfp*		KM78→SS4629 d
SS6045	*+*	+	*+*	*+*	*cat*	*+*	+	*argA::Tn10*	KM78→SS5446 d
SS6048	*kan*	*1403*	*4142*	*cat*	*cat*		Ω*gfp*	*argA::Tn10*	SS6045→SS5312 c
SS6080	*+*	+	*+*	*cat*	*+*	*+*	Ω*gfp*		SS4959→SS996 d
SS6088	*kan*	*1403*	*+*	*+*	*+*	*+*	Ω*gfp*		SS6087→SS996 i
SS6094	*kan*	+	*4142*	*+*	*+*	*+*	Ω*gfp*	*del*(*xthA*)*200::frt*	SS6009→SS4857 i

a JC13509 has the following genotype: *sulB103 lacMS286 φ 80dIIlacBK1 argE3 hi-4 thi-1 xyl-5 mtl-1 rpsL31 tsx*. The *lacMS286φ80dIIlacBK1* code for two partial non-overlapping deletions of the *lac* operon [73], [74].

b Select for Kan^R^ and then screen for other marker phenotypically or by PCR.

c Select for Tet^R^ and then screen for other marker phenotypically or by PCR.

d Select for Cat^R^ and then screen for other marker phenotypically or by PCR.

e Select for Amp^R^.

f Select for AlaS^+^.

g This deletion allele was created by first transducing the kan resistant derivative from the Kieo collection into the strain as indicated in the reference column. pLH29, carrying the *flp* gene, was then introduced and Kan sensitive derivatives were screened ([75].

h *recX::cat* was amplified with prSJS748,749 using pACYC184 (New England Biolabs) as a template. *recX::cat* was transferred to the chromosome using the *exo-bet* method [76] next to the *recA* allele indicated. This original combination of mutants were named and saved as the strain indicated as the donor in this cross.

i These *recAo* or *recA* mutations were first constructed on a plasmid as described in the Materials and Methods. They were then transferred to the chromosome using the method of Datsenko and Wanner [76] using a strain that was *lexA3 malE::Tn10* in a JC13509 background with pKD46 encoding *exo* and *bet*. This original combination of mutants were named and saved as the strain indicated as the donor in this cross.

j Full notation for *ygaD* mutation is *ygaD1::kan* .Full notation for *recX* mutation is *del(recX)4166::cat*. Full notation for *recBCD* mutation is *del(recBCD)::cat*. Full notation for Ω*gfp* mutation is *Δattλ::sulApΩgfp-mut2*.

**Table 3:** Oligonucleotide primers used in this work.

Name	DNA sequence (5′ to 3′)
prSJS453	GAAATCTACGGACCGGAATCTTCCGG
prSJS469	ATAGTTCTTTCCTGTACATAACC
prSJS515	CGAGACGAACAGAGGCGTAGTACTTCAGCGCGTTACC
prSJS516	GGTAACGCGCTGAAGTACTACGCCTCTGTTCGTCTCG
prSJS748	TTGTAAGGATATGCCATGACAGAATCAACATCCCGTCGCCCGGCATATGCGGCGAAAATGAGACGTTGATC
prSJS749	GGAAGTAAAATACCGTATGCGTTCAGTCGGCAAAATTTCGCCAAATCTCCTCAGGCGTAGCACCAGGCG

### Constructions of *recA* mutants

The *ygaD1::kan recAo1403 recA4142* mutant was initially constructed on a plasmid using cross-over PCR. The two fragments to be recombined were amplified using prSJS453,515 and prSJS516,469 with pJN3 (a derivative of pJC869 with *recA-gfp* substituted for *recA* [24]) as the template DNA. These fragments were then combined by standard cross-over PCR protocols and the resulting DNA fragment was cloned into the TA topo cloning vector, pCR2.1 (Invitrogen). This plasmid was called pSJS1354. To combine *recA4142* with *recAo1403*, pSJS1354 and pSJS1472 (plasmid containing the *ygaD1::kan recAo1403*,*4136::gfp-90*1; [24]) were restricted *Pme*I and *Blp*I. The appropriate fragments were isolated, mixed and treated with DNA ligase. The resulting plasmid, pSJS1483 was restricted with *Bam*HI and *Blp*I. The fragment was isolated and transferred to the chromosome using the *exo-bet* method as mentioned above. The resulting strain was called SS4973.

To create *ygaD1::kan recA4142*, pSJS1483 and pSJS1373 were restricted with *Xcm*I. The appropriate fragments were isolated, mixed and treated with DNA ligase to produce a plasmid containing *ygaD1::kan recA4142*,*4136::gfp-901*. This plasmid was called pNR115. pNR115 was then restricted with *Bam*HI and *Pme*I and transferred to the chromosome using the above method. The resulting strain was called SS6009.

To create *ygaD1::kan recAo1403*, pSJS1483 was restricted with *Bam*HI and *Blp*I. The *ygaD1::kan recAo1403* fragment was isolated and transferred to the chromosome using the above method. The resulting strain was called SS6087.

It should be noted in the above constructions that all alleles that were initially created by PCR protocols were subjected to DNA sequence analysis to verify the sequence.

### Preparation of Cells for Microscopy

Cultures were grown in 56/2 minimal medium or LB rich medium until mid-log phase (OD_600_ = 0.3–0.4) where appropriate. Cells were concentrated 10-fold in 56/2 buffer and mixed with an equal volume of reference beads (In-Speck, Molecular Probes). Approximately 1 µl of this mixture was loaded onto fresh agarose pads and a cover slip was applied. The agarose pads were prepared using a protocol from P. Levin [70]. Briefly, 50 µl of molten 1% agarose was loaded into the space between two parallel strips of tape on the surface of a siliconized glass plate. A clean microscope slide was pressed over the agarose creating a thin pad in between the strips of tape. The slide was inverted and cells were placed onto the surface and covered with a 22 mm^2^ coverglass.

### Microscopy and measurements

This has been described in detail with examples elsewhere [22]. Briefly, microscopy was carried out by using an epifluorescent Nikon E600 microscope. An ORCA-ER-cooled CCD camera (Hamamatsu) and Openlab software (Improvision) were used for all image acquisition and processing. Image acquisition parameters were as the following: the exposure time was 100–250 ms using a neutral single ND4 filter. Approximately nine fields (three on three different days) containing calibration beads were photographed. A phase-contrast and a fluorescent image of each field were taken. Openlab 5.0 and Volocity 4.0 software (Improvision, Inc.) were to measure the amount of fluorescence and cell size in individual cells. Calibration of the fluorescence intensity was set by calibration beads {InSpeck Green (505/515) Microscope Image Intensity Calibration Kit 2.5 µm I-7219 from Molecular Probes}. The relative intensity value of an individual cell is calculated from dividing the average calibrated pixel value of a particular cell by average calibrated pixel value of a strain containing *Δattλ::sulApΩgfp-mut2* cell (typically SS996).
